# Cardiac function modulation depends on the A‐kinase anchoring protein complex

**DOI:** 10.1111/jcmm.14659

**Published:** 2019-09-11

**Authors:** Yan‐Rong Zhu, Xiao‐Xin Jiang, Yaguo Zheng, Jing Xiong, Dongping Wei, Dai‐Min Zhang

**Affiliations:** ^1^ Department of Cardiology Nanjing First Hospital Nanjing Medical University Nanjing China; ^2^ Department of Pharmacology Nanjing Medical University Nanjing China; ^3^ Department of Oncology Nanjing First Hospital Nanjing Medical University Nanjing China

**Keywords:** A‐kinase anchoring proteins, arrhythmia, calmodulin, cardiomyocytes, hypertrophy, large‐conductance Ca^2+^‐activated K^+^ channels, sudden cardiac death

## Abstract

The A‐kinase anchoring proteins (AKAPs) are a group of structurally diverse proteins identified in various species and tissues. These proteins are able to anchor protein kinase and other signalling proteins to regulate cardiac function. Acting as a scaffold protein, AKAPs ensure specificity in signal transduction by enzymes close to their appropriate effectors and substrates. Over the decades, more than 70 different AKAPs have been discovered. Accumulative evidence indicates that AKAPs play crucial roles in the functional regulation of cardiac diseases, including cardiac hypertrophy, myofibre contractility dysfunction and arrhythmias. By anchoring different partner proteins (PKA, PKC, PKD and LTCCs), AKAPs take part in different regulatory pathways to function as regulators in the heart, and a damaged structure can influence the activities of these complexes. In this review, we highlight recent advances in AKAP‐associated protein complexes, focusing on local signalling events that are perturbed in cardiac diseases and their roles in interacting with ion channels and their regulatory molecules. These new findings suggest that AKAPs might have potential therapeutic value in patients with cardiac diseases, particularly malignant rhythm.

## 1 INTRODUCTION

A‐kinase anchoring proteins (AKAPs) have been identified in a number of species and tissues and are related to the composition of a wide variety of complexes implicated in different signalling cascades. AKAPs are distinguished by their ability to bind cyclic adenosine monophosphate (cAMP)‐dependent protein kinase A (PKA) as well as other signalling enzymes at focal points within the cell to ensure the integration and processing of multiple signalling pathways.^1^ Additional signalling proteins including adenylyl cyclases (ACs), phosphodiesterases (PDEs), protein kinases, phosphatases, GTPases and ion channels locate on the anchoring protein. AKAPs recruit these signalling molecules and form multifunctional complexes to generate protein‐protein interactions.^2^, ^3^, ^4^ AKAPs have been traditionally named on the basis of their apparent molecular weight, whereas in different species, there is disparity in the naming of the same anchoring protein. Hence, the Human Genome Organisation (HUGO) gives approval for the nomenclature of the AKAPs.^1^

**Figure 1:**
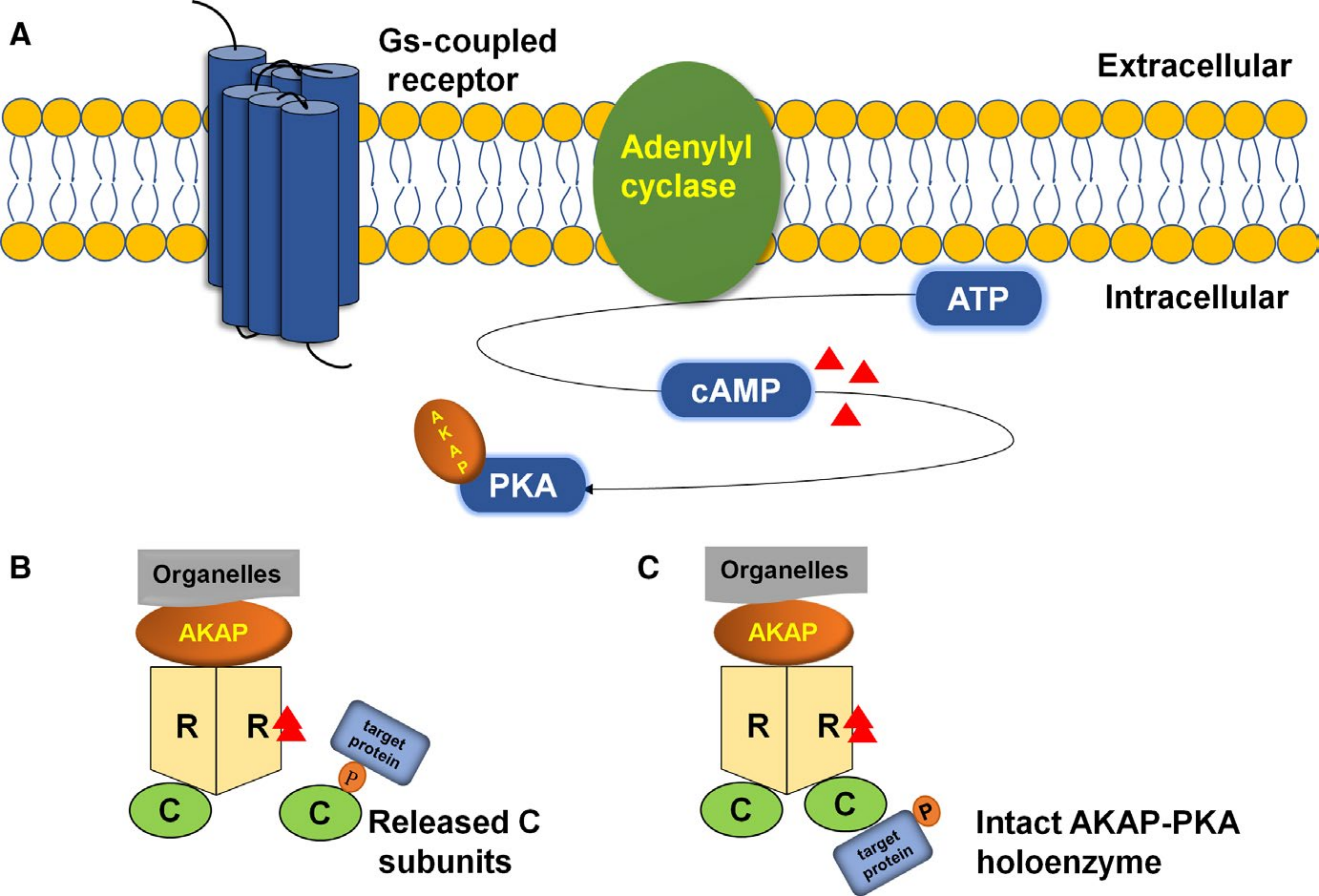
A, Stimulating GPCRs promotes signal transduction through the cAMP pathway via specific extracellular ligands leading to the activation of AC, which converts ATP into cAMP. AKAP anchors in PKA. B, cAMP binding to the R subunits of PKA increases, and the active catalytic subunits are released to phosphorylate their targets. C, This compact state may provide for the phosphorylation of associated target proteins

Cyclic AMP is a widespread intracellular second messenger that regulates numerous physiological and pathological processes. The concentration and signalling of cAMP are tightly controlled and co‐ordinated through the involvement of molecular machinery co‐ordinating the spatial and temporal processes of localized cAMP signalling events.^5^, ^6^ Stimulating G protein‐coupled receptors (GPCRs), such as β adrenoceptor, by interacting with α subunit of G_s_ protein (α_s_) promotes signal transduction through the cAMP pathway via specific extracellular ligands, leading to the activation of most ACs, which convert ATP into cAMP. Through the generation of cAMP following AC activation, cAMP‐dependent PKA is activated, and ligands that stimulate GPCRs coupled to G_i_ can inhibit AC activity. Furthermore, cAMP may be degraded by PDEs^7^, ^8^, ^9^, ^10^ (see Figure 1A).

The PKA holoenzyme is a heterotetramer consisting of two regulatory (R) subunits that maintain two catalytic (C) subunits in an inhibited state.^11^ The holoenzyme can dissociate into a regulatory subunit dimer (each monomer binds two cAMP) and two free but active catalytic subunits when binding four molecules of cAMP.^12^ There are three genes for the C subunit gene products, including Cα, Cβ and Cγ. The R genes have been divided into four different types: RIα, RIβ, RIIα and RIIβ.^13^ Cα1, Cα2 and Cα3 are contained in Cα isoforms. Cα1 exists in a wide variety of human tissues; Cα2 is mainly expressed in sperm cells; and the expression of Cα3 remains to be elucidated. The Cβ isoform has been found in human tissues, and the function of the Cγ isoform, which is expressed in testis tissue, remains unclear.^14^, ^15^ The four PKA R subunit isoforms share a universal domain organization containing the N‐terminal dimerization/docking (D/D) domain, a linker including the inhibitor site, and two consecutive cAMP‐binding domains. Differs in cAMP responsiveness and subcellular localization show that RI isoforms are predominantly diffuse in the cytoplasm and are more sensitive to cAMP signalling, whereas the RII isoforms are more localized in cells and less responsive to cAMP signalling.^16^ The RI subunits have a pseudosubstrate binding site, and the RII subunits are not only substrates but also inhibitors of the C subunit. However, the phosphorylated RII dimer does not dissociate from the C subunits in the absence of cAMP. The R subunits are tightly bound to the C subunits, thereby preventing the C subunit from interacting with external protein substrates. The cAMP‐binding domain allows the cells to turn the second messenger cAMP signal into a biological response. Therefore, when the structure of PKA is changed, cAMP‐dependent activation is decreased.^12^, ^17^, ^18^, ^19^ AKAPs are a family of functionally related proteins that interact with the regulatory subunits of the PKA holoenzyme. Through interaction between the hydrophobic pocket of PKA and the 14‐18 amino acid amphipathic helix region of AKAPs, AKAPs anchor the R subunit dimer D/D domain, and AKAPs are responsible for anchoring the two R subunits specifically. Although some AKAPs show specificity for RI and RII subunits, most AKAPs tend to show more specificity for the RII subunit than that for the RI subunit.^3^, ^20^ The RIIα D/D domain can accommodate various side chains at numerous positions of the AKAP peptide; the flexible N terminus of the D/D domain is the crucial one. At many cellular microdomains, cAMP signalling is amplified by facilitating PKA interactions with many AKAPs, which results from this kind plasticity of the D/D domain.^21^ Moreover, spatially restricted activation of PKA is guaranteed by the binding of this kinase with AKAPs^21^, ^22^, ^23^, ^24^ (see Figure 1B). In contrast, a recent study has shown that even local cAMP production stimulates kinase activity, and AKAP79:2RII:2C assemblies remain intact, which means AKAP‐PKA holoenzyme assemblies remain intact (see Figure 1C). cAMP production in response to physiological effectors of GPCR signalling appears not to promote catalytic subunit release from anchored PKA holoenzymes.^25^ But this result has been challenged recently by Gray's group,^26^ which proved that catalytic subunits are released from regulatory subunits by cAMP, and during cAMP activation, tether to R subunits does not restrict C subunit activity. These views remain controversial and have yet to be explored.

Over the decades, more than 70 different AKAPs have been discovered in various cells, and accumulating evidence has indicated that several AKAPs play key roles in modulating multiple signalling pathways in the vasculature and in the heart. By co‐ordinating signalling pathways, AKAPs modulate the physiological and pathological function of cardiomyocytes and endothelial and smooth muscle cells, thereby influencing vascular and cardiac function (Table 1). AKAPs can function in the heart to influence contractility, action potential, arrhythmias, hypoxia adaptation, heart failure and hypertrophy.^2^, ^27^, ^28^, ^29^, ^30^, ^31^, ^32^ In this review, we will provide an overview of recent results describing the functional regulation of AKAPs in cardiac pathophysiology.

**Table 1:** Characterization of AKAPs in cardiomyocytes

Function	Gene Name	Alternative Name	Binding Partners	Intracellular Localization	References
Pro‐hypertrophic	AKAP6	mAKAP	PKAII, PDE4D3, AC5, RyR2, CaNA, PP2A, NFATc, ERK5, MEK5, Epac1, Rap1, Siah2, PDK1, RSK3, NCX1, nesprin‐1α,	Nuclear envelope	1, 2, 38, 39, 40, 41, 42, 43, 44, 45, 46, 96, 97, 98
	AKAP13	AKAP‐Lbc, Ht31	PKA RII, RhoA, Actin, PKC, PKD, KSR1, Raf, MEK1/2, ERK1/2, PKNα	Cytoskeleton	1, 31, 48, 49, 50, 51, 52, 53, 54, 55, 56, 57, 58, 59, 60, 99, 100
Anti‐hypertrophic	AKAP7	AKAP15, AKAP18	PKAII, L‐type Ca^2+^ channel, phospholamban, PP1, inhibitor 1	Plasma membrane, endoplasmic reticulum	1, 4, 59, 60, 61, 62, 63, 101
	AKAP1	D‐AKAP1, AKAP121, AKAP149	PKAI and II, PKCα, Src, RSK1, PP1, PP2A, CaN, PTPD1, Lfc	Mitochondria, nuclear envelope, endoplasmic reticulum	1, 2, 35, 36, 37, 102, 103, 104
	AKAP5	AKAP79, AKAP150	PKAII, PKC, CaN, KCNQ2, L‐type Ca^2+^ channel, β‐AR, AC5 and AC‐6, SAP97, caveolin‐3	Plasma membrane, T tubules	1, 2, 3, 22, 28, 65, 66, 67, 84, 85, 86, 87, 105, 106
Contractility	AKAP5	AKAP79, AKAP150	PKAII, PKC, CaN, KCNQ2, L‐type Ca^2+^ channel, β‐AR, AC5 and AC‐6, SAP97, caveolin‐3	Plasma membrane, T tubules	1, 2, 3, 22, 28, 65, 66, 67, 84, 85, 86, 87, 105, 106
	AKAP12	Gravin, AKAP250	PKA RII, β‐AR, PKC, PDE4D, Src	Plasma membrane	1, 2, 80, 81, 82, 83, 107, 108
Arrhythmias	AKAP9	Yotiao, AKAP350, AKAP450	PKAII, PP1, PP2A, PKC, PKN1, kinase 1, AC, PDE4D3, KCNQ1, CLIC	Plasma membrane, Golgi, centrosome	1, 2, 3, 84, 85, 86, 87, 88, 109, 110
	AKAP5	AKAP79, AKAP150	PKAII, PKC, CaN, KCNQ2, L‐type Ca^2+^ channel, β‐AR, AC5 and AC‐6, SAP97, caveolin‐3	Plasma membrane, T tubules	1, 2, 3, 22, 28, 65, 66, 67, 84, 85, 86, 87, 105, 106

## 2 CARDIAC HYPERTROPHY

### 2.1 D‐AKAP1

D‐AKAP1, which means a dual‐specificity A‐kinase anchoring protein, binds to both the RI and RII subunits of PKA.^33^ Several D‐AKAP1 isoforms or homologues were identified in various species. These isoforms include mouse AKAP121, rat AKAP121 and human AKAP149.^34^ It has been shown that down‐regulation of D‐AKAP1 is related to oxidative stress, mitochondrial dysfunction, cardiomyocyte hypertrophy and apoptosis.^35^, ^36^, ^37^

Previous studies have shown that knockdown of D‐AKAP1 induces, rather than inhibits, hypertrophy. In contrast, overexpression of D‐AKAP1 has the opposite effect on cell size. On one hand, cell size is reduced by increased D‐AKAP1 expression. The effect of the hypertrophic adrenergic agonist isoproterenol is inhibited. The result of D‐AKAP1 knockdown on hypertrophy is mediated by the activation of the calcineurin (CaN)/nuclear factor of activated T cell (NFATc) pathway, as shown by alterations in intracellular NFATc3 localization. Small hairpin RNA (shRNA) experiments have been performed to show that D‐AKAP1 knockdown induces NFATc3 dephosphorylation and translocation to the nucleus, resulting in hypertrophy.^35^

In addition, other studies demonstrated that D‐AKAP1 is an important regulator of mitochondrial function and cell survival, and thus, D‐AKAP1 down‐regulation may represent an important event in the development of cardiac dysfunction. Displacement of D‐AKAP1 from mitochondria is closely related to increased reactive oxygen species (ROS) generation, and ROS production induces D‐AKAP1 degradation. Accumulation of ROS also promotes cardiomyocyte apoptosis. This is an important observation because it suggests that improper cAMP signalling can spill over into mitochondrial regulatory pathways, connecting cardiomyocyte survival and oxidative stress.^36^

### 2.2 mAKAP

The scaffolding protein muscle‐selective AKAP (mAKAP), also known as AKAP6,^38^ is a PKA‐anchoring partner that is expressed in the brain, heart and skeletal muscle. α and β are two alternatively spliced forms of mAKAP, which is required in cardiac myocytes for the induction of cardiac hypertrophy by transverse aortic constriction and isoproterenol infusion.^39^, ^40^ However, mAKAP‐β, mainly expressed in heart and skeletal muscle, plays a crucial role in myoblast differentiation, myotube formation and muscle regeneration.^38^, ^41^ The classical view is that mAKAP complex anchoring extracellular‐regulated protein kinases 5 (ERK5) can induce cardiac hypertrophy.^42^ During the past few years, some literature has revealed several novel signalling pathways by which mAKAP regulates cardiac hypertrophy.^39^, ^40^, ^41^, ^42^, ^43^, ^44^, ^45^

First, phospholipase Cε (PLCε) scaffolded to mAKAP is a multifunctional enzyme implicated in cardiovascular, pancreatic and inflammatory functions. Evidence shows that PLCε generates second messengers at the nuclear envelope that are required for hypertrophy, and phosphatidylinositol 4‐phosphate (PI4P) is a perinuclear substrate in the Golgi apparatus for mAKAP‐scaffolded PLCε.^43^, ^44^ PI4P, together with PLCε, is a substrate for mammalian PLC isoforms. Activation of mAKAP‐scaffolded PLCε is directly involved in perinuclear PI4P depletion, which means that, as a perinuclear enzyme, PLCε can hydrolyse PI4P to produce diacylglycerol (DAG). Notably, cardiac hypertrophy development is significantly reduced after the cardiac‐specific deletion of PLCε. This strongly suggests that mAKAP‐PLCε signalling in cardiac myocytes is important for hypertrophy development. Furthermore, the neonatal myocyte analysis of PLCε function is largely relevant to the function of the whole heart.^44^

In addition, the scaffolding protein mAKAP organizes a calcineurin/myocyte enhancer factor 2 (MEF2) signalling complex in myocytes to regulate gene transcription. In the stressed heart, MEF2 is significant for the transactivation of hypertrophic gene transcription.^45^, ^46^ A laboratory used primary neonatal rat cardiac myocytes transfected with expression plasmids for either control mCherry or mCherry‐CaNBD and then stimulated the cells for two days with norepinephrine, which is a type of adrenergic agonist that can increase the cross‐sectional area of cells. By measuring the cellular cross‐sectional area on images, it was found that there was no distinct difference in size between treated and untreated myocytes expressing mCherry‐CaNBD. Meanwhile, the expression of atrial natriuretic factor (ANF), a marker for hypertrophy encoded by the MEF2‐transactivated Nppa gene, was down‐regulated in mCherry‐CaNBD‐expressing myocytes after adrenergic stimulation. Taken together, these data suggest that calcineurin binding to mAKAP is required for the induction of cardiac hypertrophy and that this event is mediated by MEF2.^46^

Additionally, mAKAP‐β contributes to the orchestration of Ca^2+^‐dependent signalling transduction. During states of elevated sympathetic stimulation, PKA‐catalysed ryanodine receptor Ca^2+^ release channel (RyR2) phosphorylation could increase local Ca^2+^ release with the participation of mAKAP‐β. Ca^2+^ is released to induce sarcomeric contraction, and mAKAP‐β complexes may connect contractility to the induction of hypertrophy.^38^ Lee et al further studied skeletal myoblast differentiation and muscle regeneration based on mAKAP. mAKAP knockdown was shown to markedly impede the formation of myotubes and decrease myoblast differentiation and skeletal muscle regeneration.^41^

### 2.3 AKAP‐Lbc

AKAP‐Lbc (also known as AKAP13 and Ht31^47^) is a Rho‐specific guanine nucleotide exchange factor inside cells, and it functions as a scaffolding protein to co‐ordinate the Rho signalling pathway. AKAP‐Lbc not only anchors PKA but can also activate Rho.^48^ Diviani's group has undertaken a number of fundamental studies on AKAP‐Lbc, and they identified AKAP‐Lbc as the first Rho‐guanine nucleotide exchange factor (GEF) involved in signalling pathways leading to cardiomyocyte hypertrophy by activating RhoA and transducing hypertrophic signals downstream of α1‐adrenergic receptors (ARs).^49^ It has been demonstrated that AKAP‐Lbc is up‐regulated in human hypertrophic cardiomyopathy.^50^ However, AKAP‐Lbc assembles a macromolecular signalling complex to co‐ordinate the activity of transduction enzymes, which has a direct impact on compensatory hypertrophy and maintenance of cardiac function during the early‐phase of cardiac remodelling.^31^

AKAP‐Lbc‐ΔPKD1 is a truncated form of AKAP‐Lbc that is unable to bind PKD1. Because of the deletion of the PKD1 binding domain on AKAP‐Lbc, AKAP‐Lbc‐ΔPKD mice exhibit reduced myocyte hypertrophy with increased cardiac extracellular collagen synthesis and apoptosis in response to transaortic constriction (TAC)–induced pressure overload or angiotensin (AT‐II) and phenylephrine (PE) infusion.^51^ Furthermore, AKAP‐Lbc‐∆PKD1 mice display an altered cardiac transcriptional response to TAC‐induced pressure overload, which means AKAP‐Lbc‐PKD1 signalling is critical for transcriptional regulation during the development of compensatory hypertrophy.^52^ However, the AKAP‐Lbc/PKD1 complex has been shown to prevent mitochondrial dysfunction and cardiomyocyte death induced by doxorubicin. As a molecular platform, AKAP‐Lbc co‐ordinates protective signals preventing DOX‐induced cardiomyocyte toxicity. Stimulation of α_1_‐adrenergic receptors (ARs) contributes to the activation of AKAP‐Lbc‐anchored PKD_1_, and in DOX‐treated cardiomyocytes, two anti‐apoptotic pathways are activated to enhance the expression of Bcl2 and inhibit the mitochondrial translocation of the pro‐apoptotic protein Bax. AKAP‐Lbc/PKD1 complex functions to prevent mitochondrial dysfunction and cardiomyocyte death induced by DOX.^53^

**Figure 2:**
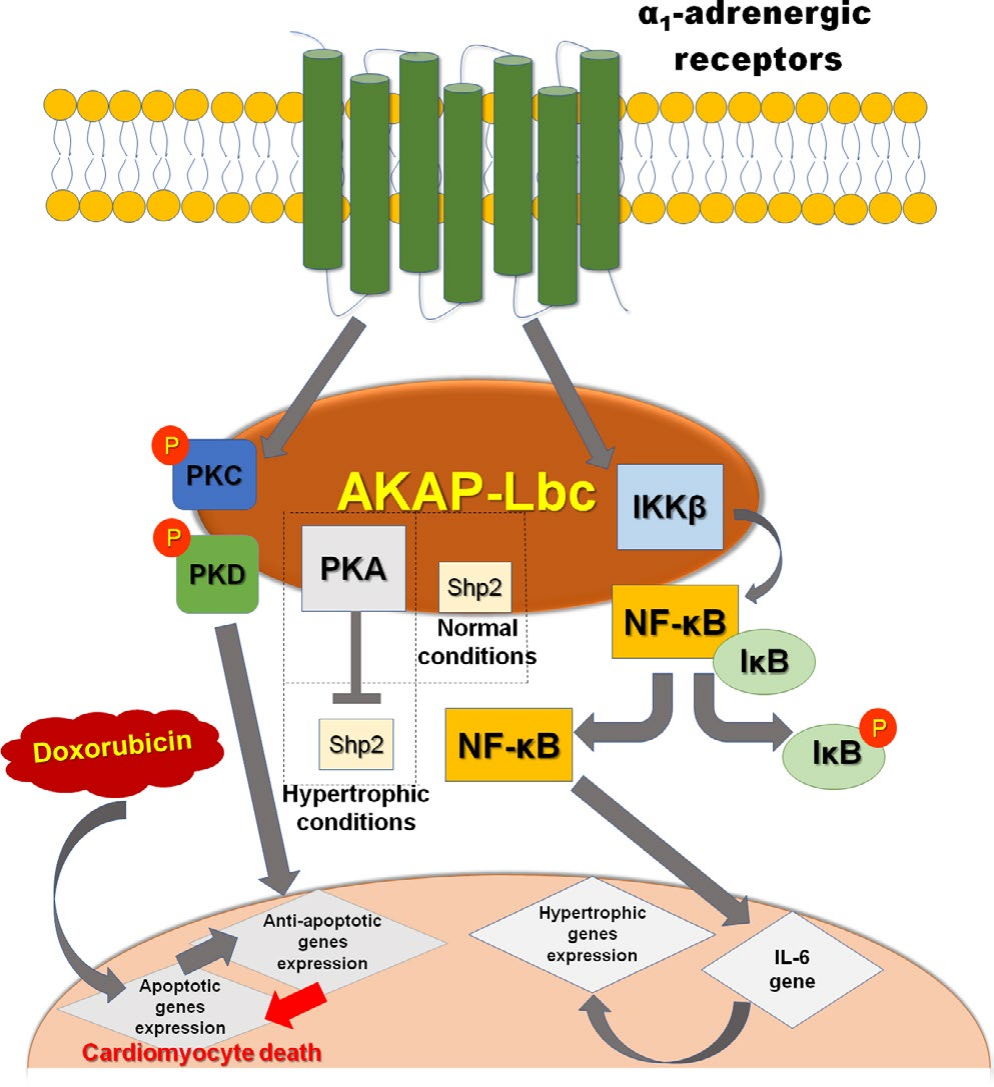
In cardiomyocytes, AKAP‐Lbc mediates IKKβ activation after stimulation of α_1_‐AR. Activated IKKβ leads to NF‐κB‐dependent production of IL‐6, which in turn engages signalling pathways controlling the transcription of cardiomyocyte hypertrophic genes. AKAP‐Lbc assembles a signalling complex composed of PKA and Shp2 in cardiac myocytes. Some conditions lead to PKA activation, thereby promoting inhibition of Shp2 activity, which may contribute to the induction of cardiac hypertrophy, and the AKAP‐Lbc/PKD signalling complex mediates protection against doxorubicin (DOX)‐induced cardiomyocyte death

The tyrosine phosphatase Shp2 is a component of the A‐kinase anchoring protein (AKAP)‐Lbc complex, and the interaction of AKAP‐Lbc and Shp2 inside cells is complicated. Shp2 is a PKA substrate; Shp2 is phosphorylated by PKA in cardiac myocytes in response to isoproterenol stimulation. At the same time, AKAP‐Lbc plays an important role in the regulation of Shp2 activity by facilitating the phosphorylation of Shp2 by PKA.^54^ Two key amino acids in Shp2, Thr‐73 and Ser‐189 are phosphorylated by PKA.^55^ In summary, chronic activation of PKA in the hypertrophic heart promotes the inhibition of Shp2 activity associated with AKAP‐Lbc^54^ (see Figure 2).

IκB is an inhibitor of the transcription factor NF‐κB, which is a mediator of the growth responses induced by a variety of pro‐hypertrophic agonists.^56^ NF‐κB is recognized as a key transcription factor mediating cardiac hypertrophy.^57^ The inhibitor of IκB kinase (IKK) complex (IKKα, IKKβ and IKKγ) contributes to the phosphorylation of IκB under stimulation.^58^, ^59^ AKAP‐Lbc promotes the activation of anchored IKKβ, which in turn results in the phosphorylation and degradation of IκB and activation of NF‐κB. Finally, activated NF‐κB induces transcription of the IL‐6 gene and subsequent stimulation of IL‐6‐mediated pathways to control foetal gene transcription and cardiomyocyte hypertrophy^60^ (Figure 2).

### 2.4 AKAP15

AKAP15 (also known as AKAP7 and AKAP18^61^), which anchors PKA to calcium channels, is a family of alternatively spliced isoforms (α, β, γ and δ) that are known to play a role in cardiac L‐type calcium dynamics.^62^ A transgenic mouse with destructed AKAP15/L‐type Ca^2+^ channel (LTCCs) binding is not sensitive to cAMP stimulation, and the mice also suffer from cardiac hypertrophy.^4^ AKAP15 directly interacts with the distal C terminus of the cardiac CaV1.2 channel via a leucine zipper‐like motif, and AKAP15 facilitates cardiac contraction via regulation of beta‐adrenergic (β‐AR)‐stimulated L‐type Ca^2+^ channels.^62^, ^63^, ^64^ The distal C‐terminal domain (DCT) maintains a non‐covalent interaction with the truncated CaV1.2 channel, acting as an autoinhibitor of the channel and reducing the channel's sensitivity. DCT is required for normal responses to β‐AR signalling and AKAP15 localization. Deletion of the DCT induces cardiac hypertrophy, possibly as a result of impairment of regulation of the peripheral vasculature, such as increased peripheral vascular resistance. CaV1.2 channels without DCT cannot be regulated by the β‐AR/PKA signalling pathway and cannot support normal expression and localization of AKAP15. This result underscores the importance of AKAP15 in normal excitation‐contraction coupling and suggests that AKAP15 plays a role as an inhibitor of cardiac hypertrophy.^65^

### 2.5 AKAP5

Although the expression of AKAP5 (murine AKAP150, human AKAP79^61^) in the heart is low, it is widely expressed in the periphery and plays a major role in forming discrete signalling networks. AKAP5 is able to bind and inactivate Ca^2+^/calmodulin‐dependent phosphatase (CaN). On this condition, CaN‐mediated cardiac hypertrophy can be inhibited.^22^, ^28^ However, endogenous CaN activity does not directly regulate cardiac Ca^2+^ channel activity in mouse myocytes. AKAP5 mice with significantly diminished endogenous CaN activity can retain normal myocyte size.^66^ Cardiac β‐ARs are key regulators of cardiac size. AKAP5 is a key regulator of myocardial signalling by β‐ARs. Deletion of AKAP5 was associated with significant cardiac hypertrophy.^28^, ^67^ Because deletion of AKAP5 prevented the recycling of internal β_1_‐AR, the influence did not include the internalization of β_1_‐AR in mouse cardiac myocytes.^68^

## 3 MYOFIBRE CONTRACTILITY DYSFUNCTION

### 3.1 AKAP79/150

AKAP79/150 interacts with PKA, protein kinase C(PKC), Ca^2+^/calmodulin‐dependent phosphatase (CaN), calmodulin (CaM) and other signalling molecules to regulate vascular tone and blood pressure.^28^, ^29^, ^30^, ^69^, ^70^

During hyperglycaemia and diabetes, AKAP79/150 is reported to contribute to enhancing vascular tone through facilitating large‐conductance Ca^2+^‐activated K^+^ (BK) channel remodelling. AKAP150 anchors CaN and mediates nuclear factor of activated T cell c3 (NFATc3) activation and the transcriptional suppression of regulatory BK‐β_1_ subunit during diabetes induced by glucose,^29^ and the BK‐β_1_ subunit is a crucial regulatory factor of vascular tone.^71^ In conclusion, anchoring of calcineurin by AKAP150 is required for BK channel impairment during hyperglycaemia and diabetes, which promotes enhanced vascular tone.^29^

In addition, hypercontractility of arterial myocytes and enhanced vascular tone during diabetes are attributed to the effects of increased glucose on L‐type CaV1.2 channels.^72^ α_1_C is a subpopulation of the CaV1.2 channel pore‐forming subunit, and Ser^1928^ is a highly conserved PKA consensus phosphorylation site located within the intracellular C terminus of α_1_C. As a key molecular signalling event underlying the potentiation of Ca_V_1.2 channel activity and vasoconstriction upon acute increases in extracellular D‐glucose and diabetes, the AKAP‐dependent, PKA‐meditated phosphorylation of α_1_C at Ser^1928^ plays a vital role in this progression.^73^

The expression of transient receptor potential vanilloid 4 (TRPV4) channels is comprehensive, and they belong to a kind of Ca^2+^‐permeable, non‐selective cation channel.^74^ Contractile function is closely tied to TRPV4 channels in that cardiomyocyte TRPV4 is a novel mediator of enhanced contractile function early in ischaemia‐reperfusion.^75^ Endothelial impairment can influence the regulation of vascular tone, and endothelial cells (ECs) are assumed to be an important regulator of vasodilatory function. Stimulating some receptors on ECs excites TRPV4 channels, which are localized at myoendothelial projections (MEPs). The PKC‐anchoring protein AKAP79/150 mainly localizes to MEPs, which contributes to the opening of TRPV4 and enhances local Ca^2+^ influx. In contrast, in hypertension, this molecular assembly is disrupted.^76^ However, in the sarcolemma of arterial myocytes, the PKCα‐associated, AKAP150‐dependent modulation of TRPV4 channels relies on the distance between these two proteins.^77^

### 3.2 Gravin

Beta‐adrenergic receptors (β‐ARs), and especially β_2_‐AR, are identified as significant regulators of cardiac contractility by activating PKA.^78^ Gravin, also known as AKAP12 and AKAP250, has the ability to bind β_2_‐AR.^79^ Therefore, gravin plays an indispensable role in the β‐AR‐mediated regulation of cardiac contractility.^80^

In one experiment, isoproterenol (ISO) was applied in wild‐type (WT) and gravin mutant (gravin‐t/t) mice to detect cardiac contractility, and it was found that, at diastole, there was no obvious difference between WT and gravin‐t/t mice. However, at systole, left ventricular internal dimensions (LVID) were decreased in the gravin‐t/t mice compared with WT mice. Moreover, cardiomyocytes isolated from gravin‐t/t mice had enhanced cardiomyocyte contractility in the presence of a proportionally lower diastolic baseline and maximum height of intracellular Ca^2+^ transients. These results indicated that gravin is a key factor in the desensitization/resensitization cycle of β_2_‐AR. The signalling mechanism resulting from disruption of gravin's scaffold is such that when the gravin gene is mutant in mice, the baseline cardiac function is increased, and contractility is enhanced in response to acute β‐AR stimulation. At the same time, the phosphorylation of β_2_‐AR is decreased, which in turn attenuates receptor desensitization.^80^

Li et al used right ventricles of gravin mutant (gravin‐t/t) mice to test the effect of acute β‐AR stimulation on cardiac contractility in vivo on the absence of gravin binding to β_2_‐AR, PKA and other signalling molecules. It was shown that gravin‐t/t muscles exhibited increased myofilament Ca^2+^ responsiveness while maintaining their ability to release Ca^2+^ from the sarcoplasmic reticulum (SR). The phenomenon revealed that, besides serving as a scaffolding protein, gravin functions as a regulator of myofilament Ca^2+^ sensitivity. It is obvious that gravin is an important regulator of cardiac contraction via increasing myofilament sensitivity to Ca^2+^.^81^

## 4 CARDIAC ARRHYTHMIAS

### 4.1 Yotiao

Yotiao is a splice variant of the AKAP9 gene and is present on the plasma membrane. Yotiao displays specificity among AC isoforms and interacts with AC 1, 2, 3 and 9. In addition, Yotiao can co‐ordinate the assembly of the I_Ks _signalling complex.^82^, ^83^

Long QT syndrome (LQTS) is a heritable arrhythmia syndrome.^84^ Previously, it was found that, in the heart, Yotiao (AKAP9) assembles with KCNQ1, which is short for I_Ks_ potassium channel subunit, to regulate cardiac action potential duration (APD). Type 1 long QT syndrome (LQT1) results from the disruption of this complex.^85^ Further experiments were performed to explore the Yotiao missense mutational site, and S1570‐Yotiao was shown to modify Yotiao/KCNQ1 interactions and PKA phosphorylation; furthermore, it also reduced the functional response of I_ks_ channels to cAMP. Therefore, it is obvious that, as an inherited mutation of an AKAP9‐encoded protein, S1570‐Yotiao is relevant to LQTS, and this finding may provide evidence for future clinical treatment.^86^

### 4.2 AKAP150

The interaction between AKAP150 and long QT syndrome 8 (LQT8) is also known as Timothy syndrome and is characterized by a single amino acid substitution (G406R) in the L‐type Ca^2+^ (Ca_V_1.2) channel.^87^ Disruption of AKAP150 improves pathological Ca_V_1.2‐LQT8 channel gating and arrhythmias and prevents hypertrophy of LQT8 hearts by decreasing Ca^2+^ influx via Ca_V_1.2‐LQT8 channels.^88^

AKAP150 is essential for sympathetic stimulation of the Ca^2+^ transient. AKAP150‐null mice showed unstable R‐R intervals and decreased LF, indicating that the tonus of the sympathetic nerves had been modified. In addition, the AKAP5‐null atrium showed a decreased contractile response to isoproterenol, which means AKAP5‐null mice exhibit a modulated sympathetic nerve response.^89^, ^90^

### 4.3 D‐AKAP2

AKAP10 (D‐AKAP2) binds with high affinity to both the RI and RII regulatory subunits of PKA, and the structure of AKAP10 consists of two tandem regulators of G protein signalling (RGS)–like homology domains followed by a 27‐residue PKA‐binding (AKB) domain and a PSD‐95/DlgA/ZO‐1(PDZ)‐binding motif at the C terminus.^91^ D‐AKAP2_AKB_ binds to the D/D domain of the R subunit, and the C‐terminal PDZ motif binds to a PDZ domain of NHERF_1_, NHERF_2_ and PDZK_1_, which serves as a bridging protein to the transporter.^92^

When AKAP10 is mutated in mice, the sensitivity of cultured cardiac cells to cholinergic vagus nerve inputs increases. This result is the same in living mice. In addition, AKAP10‐mutant mice displayed two types of spontaneous cardiac pauses. First, sinus pauses with junctional escape beats were 40 times more frequent in homozygous AKAP10‐mutant mice than in WT mice. Second, atrioventricular (AV) heart block was 15 times more frequent in homozygous AKAP10‐mutant mice. Both types of pauses were typically preceded by changes characteristic of vagus nerve activity.^93^ Łoniewska et al demonstrated a possible association between the 1936A > G AKAP10 variant and QTc in the aboriginal European newborn population.^94^

## 5 CONCLUSIONS AND PERSPECTIVES

It has become increasingly obvious that cardiac AKAP complexes have shed new light on how local signals are co‐ordinated and processed in vascular and cardiac functions. The role of protein kinase compartmentalization is critical in mediating the kinase signalling pathways and explains the development of different disease pathologies in the presence or absence of these AKAPs. All of the experiments above demonstrate the role of the AKAP signalling pathway in diseases such as cardiac hypertrophy, contractility dysfunction and arrhythmias by anchoring PKA, PKC, CaN and CaM. Furthermore, ion channels (L‐type Ca^2+^ channels, BK channels) are also closely associated with AKAPs.^73^, ^87^ On the molecular level, we believe that the implementation of new technologies related to the structural determination of large multiprotein complexes will provide new ways to understand the mechanism of how AKAP complexes function. This will help us to establish specific therapeutic approaches to AKAP‐related diseases. For example, AKAPs assemble localized signalosomes positioning relevant downstream effectors near respective substrate proteins to propagate downstream signalling; however, degradation of cAMP can halt signalling. In cardiovascular system, one family of the known cAMP receptors, the exchange proteins directly activated by cAMP (EPACs), is associated with cardiac hypertrophy. cAMP sensor EPAC‐based therapeutics represent promising alternatives for the management of cardiovascular diseases.^95^ In addition, AKAPs‐related arrhythmia‐causing mutations will help promote progress towards better therapeutic strategies, and there remains a need for specific treatment towards individuals in a genotype‐driven.

## CONFLICT OF INTEREST

The authors declare that they have no competing interest.

## AUTHORS CONTRIBUTIONS

Zhu YR and Jiang XX contributed to draft the manuscript; Zheng YG, Xiong J and Wei DP contributed to the discussion; and Zhang DM contributed to conceive and design the review, wrote and revised the manuscript.
